# Metabolism, Excretion, and Mass Balance of [^14^C]-Rezafungin in Animals and Humans

**DOI:** 10.1128/AAC.01390-21

**Published:** 2022-01-18

**Authors:** Voon Ong, Sarah Wills, Deborah Watson, Taylor Sandison, Shawn Flanagan

**Affiliations:** a Cidara Therapeutics, Inc., San Diego, California, USA; b Labcorp, Madison, Wisconsin, USA; c QPS, LLC, Newark, Delaware, USA

**Keywords:** pharmacokinetics, ADME, antifungal, radiolabel, echinocandin, pharmacokinetics, radioactivity

## Abstract

Rezafungin is a novel echinocandin being developed for treatment of candidemia and invasive candidiasis and for prevention of invasive fungal disease caused by *Candida*, Aspergillus, and Pneumocystis spp. in recipients of blood and marrow transplantation. Studies using [**^14^**C]-radiolabeled rezafungin were conducted in rats, monkeys, and humans to characterize the mass balance, excretion, and pharmacokinetics of [**^14^**C]-rezafungin and to evaluate relative amounts of rezafungin metabolites compared with parent drug. Fecal excretion was the main route of elimination in rats, monkeys, and humans. Radioactivity was primarily excreted as unchanged drug, with ≥95% average total recovery in rats (through 336 h) and monkeys (through 720 h). In humans, cumulative recovery of radioactivity through the first 17 days was 52% (38% in feces, 14% in urine) with estimated mean overall recovery through day 60 of 88.3% (73% in feces, 27% in urine). The clinical pharmacokinetics of rezafungin following a single 400-mg intravenous infusion (200 μCi of [**^14^**C]-rezafungin) were similar in plasma, plasma total radioactivity, and whole blood total radioactivity. Unchanged rezafungin represented the majority of total radioactivity in plasma, and the partitioning of total radioactivity into red blood cells was negligible. Across species, rezafungin was primarily metabolized by hydroxylation of the terphenyl, pentyl ether side chain. In these excretion/mass balance, metabolism, and PK studies, clinical observations were consistent with findings in the rat and monkey demonstrating the minimal metabolism and slow elimination of rezafungin after intravenous administration, with fecal excretion as the major route of elimination.

## INTRODUCTION

Rezafungin is a novel echinocandin that classically inhibits production of 1,3-β-d-glucan in the fungal cell wall and is distinguished by next-generation pharmacokinetics (e.g., long half-life and front-loaded plasma drug exposure, extensive distribution and penetration to sites of infection) that allow for once-weekly intravenous dosing and may confer greater antifungal efficacy (1).

Rezafungin has a broad spectrum of activity against Candida and Aspergillus species, including Candida auris and subsets of drug-resistant fungal strains, and has demonstrated treatment and prophylactic efficacy in animal models of candidiasis, aspergillosis, and Pneumocystis pneumonia (2–11).

Rezafungin has demonstrated safety and tolerability comparable to that of currently available echinocandins. Furthermore, rezafungin has demonstrated chemical and metabolic stability and no hepatotoxicity compared with anidulafungin (12, 13). The clinical database on rezafungin safety includes phase 1 trial data not available for the existing echinocandins as such evaluations were not included in their drug development; these rezafungin trials showed its lack of effect on the QT interval and low risk of DDIs with commonly co-administered drugs (14, 15). The completed Phase 2 treatment trial of rezafungin compared with caspofungin (STRIVE; NCT02734862) met its primary objective of safety. Two phase 3 trials of rezafungin are under way, one in the treatment of candidemia and invasive candidiasis (ReSTORE; NCT03667690) and one in the prevention of invasive fungal disease caused by species of Candida, Aspergillus, and Pneumocystis in recipients of blood and marrow transplantation (ReSPECT; NCT04368559).

Using non-radiolabeled rezafungin, tissue/plasma AUC_0-120h_ ratios in rats following IV administration indicated rezafungin exposures relative to plasma were comparable for highly perfused major organs and approximately 4- to 5-fold higher in kidney, lung, liver, and spleen than in plasma (16). Subsequently, additional studies using [**^14^**C]-radiolabeled rezafungin were conducted in rat, monkey, and human to characterize the mass balance, excretion, and PK of [**^14^**C]-rezafungin. Radiolabel metabolite identification and profiling was also conducted to quantify relative amounts of rezafungin metabolites compared with parent drug.

## RESULTS

### Rat excretion/mass balance.

**FIG 1:**
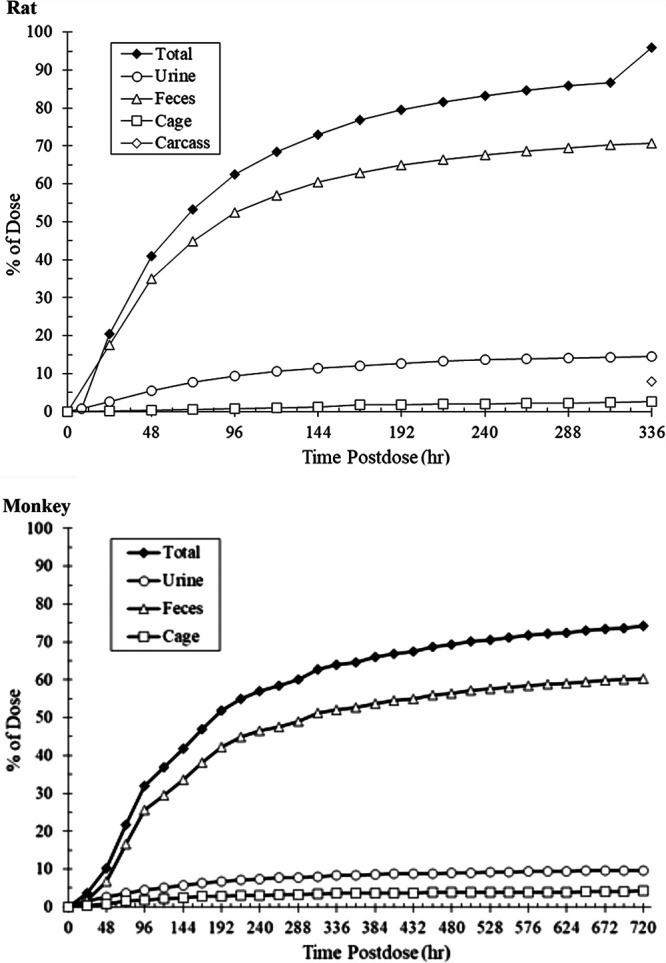
Cumulative mean recovery of radioactivity from rat (top) and monkey (bottom) collected through 30 days.

The primary route of elimination of radioactivity in intact (non-bile duct cannulated) rats was in the feces, which accounted for a mean of 70% of the administered dose. An average of 14% and 2.5% of the administered dose was recovered in the urine and cage residues, respectively, and an average of 7.9% of the administered dose was recovered in the carcass at 336 h postdose. The average total recovery of radioactivity was 95% of the administered dose as shown in Fig. 1 (top). In bile duct cannulated rats, the primary route of elimination of radioactivity after a single IV dose of [**^14^**C]-rezafungin was also in the feces (mean of 35% of the administered dose), which suggests passive diffusion of [**^14^**C]-rezafungin-derived radioactivity from the small intestine. An average of 17%, 14%, and 1.2% of the administered dose was recovered in the bile, urine, and cage residues respectively, and an average of 30% of the administered dose was recovered in the carcass. The average total recovery of radioactivity in bile duct cannulated rats was 98% of the administered dose.

### Monkey excretion/mass balance.

Excretion of [^14^C]-rezafungin derived radioactivity in urine and feces was sustained with an average total recovery at 168 h (1 week) postdose of 47.02% of the dose. The mean total recovery gradually increased per day to 65% of the dose through 360 h postdose and to 74% of the dose through 720 h postdose, as shown in Fig. 1 (bottom). Similar to the rat, the primary route of elimination of radioactivity was in the feces, which accounted for 60% of the administered dose at 720 h postdose. A total of 9.7% and 4.3% of the administered dose was recovered in the urine and cage residue, respectively.

Following a single IV 20-min infusion, maximum blood and plasma radioactivity concentrations were observed at 0.5 h (30 min) postdose, the time of the first postdose sample collection. Individual blood-to-plasma concentration ratios ranged from 0.86 to 1.7 and generally were approximately 1 across time points with a ratio of blood-to-plasma AUC also of approximately 1, suggesting that rezafungin had limited partition into the cellular fraction of whole blood.

### Human excretion/mass balance.

**(i) Subjects.** Nine healthy male subjects (average age, 41 years) received a single IV 400-mg rezafungin infusion containing 200 μCi of [^14^C]-rezafungin. Of the 9 subjects, 4 (44.4%) were Black/African American, 4 (44.4%) were White, and 1 (11.1%) was Asian. Seven (77.7%) subjects were of non–Hispanic or Latino ethnicity. The median height, weight, and BMI of subjects were 182.1 cm, 81.1 kg, and 24.1 kg/m^2^, respectively.

**(ii) Clinical safety.** A total of 13 treatment-emergent adverse events (TEAEs) were reported in 5 subjects. The most common TEAE by PT was diarrhea, occurring in two subjects; all other events occurred in a single subject. All TEAEs were mild except for a single event of moderate muscle tightness in one subject. There were no severe TEAEs. The only TEAEs considered by the investigator to be related to study treatment were a single event of mild constipation and a single event of mild infrequent bowel movements in one subject each. There were no deaths or other SAEs during the study, and no subjects withdrew from the study due to a TEAE. There were no clinically relevant treatment-related findings observed for laboratory assessments, vital signs measurements, ECGs, or physical examinations.

**(iii) Clinical pharmacokinetics.** Rezafungin concentrations and [^14^C]-rezafungin equivalents were quantifiable at all scheduled postdose collections through day 60. Ratios of whole blood to plasma total radioactivity ranged between 0.86 and 1.0 through day 60, indicating that [^14^C]-rezafungin did not predominantly partition into the cellular fraction of whole blood and remained mainly in the plasma.

Pharmacokinetic parameters (see Table 1) were similar between rezafungin in plasma, plasma total radioactivity, and whole blood total radioactivity. The rezafungin AUC accounted for the majority of the radiocarbon AUC in plasma.

**TABLE 1:** Summary of mean (SD) pharmacokinetic parameters for rezafungin, plasma total radioactivity, and whole blood total radioactivity for human subjects^*a*^

Parameter^*b*^	Plasma rezafungin	Plasma total radioactivity	Whole blood total radioactivity
AUC_0-t_^*c*^	2050 (137)	2600 (163)	2390 (175)
AUC_0-∞_^*c*^	2110 (141)	2750 (182)	2450 (200)
*C*_max_ (μg/ml)	18.9 (2.50)	18.2 (2.28)	18.5 (2.24)
*t*_max_ (hours)	1.00 (1.00, 1.00)	1.00 (1.00, 1.00)	1.00 (1.00, 1.00)
*t*_½_ (hours)	341 (42.8)	387 (44.8)	408 (39.7)
CL (liters/hour)	0.180 (0.131)	NA	NA
*V_Z_* (liters)	88.6 (12.0)	NA	NA

a AUC_0-t_ = area under the concentration-time curve from 0 to the last measurable concentration; AUC_0-∞_ = AUC extrapolated to infinity; *C*_max_ = maximum observed concentration; CL = apparent systemic clearance; max = maximum; min = minimum; SD = standard deviation; *t*_max_ = time to maximum observed concentration; *t*_½_ = terminal elimination halflife; *V_z_* = apparent volume of distribution based on the terminal elimination phase.

b Results are reported as arithmetic mean (SD), except for *t*_max_, which is reported as median (min, max).

c Units are μg×hour/ml for rezafungin, μg equivalent×hour/ml for plasma and whole blood total radioactivity.

**(iv) Mass balance.** Radioactivity was primarily excreted in feces during the in-clinic portion (first 17 days) of the study; cumulative recovery of radioactivity from excreta collected through the first 17 days was 52% (38% in feces, 14% in urine), highlighting the slow overall elimination of rezafungin. Based on interpolated data (using data from the subjects’ return visits to the CRU on day 29 and day 60), it was estimated that the majority of the dose (an overall mean estimate of 88.3%) would have been recovered had the subjects been continuously confined to the clinic through day 60. Approximately 73% of this total was recovered in feces while the total in urine was 27%, indicating that elimination is primarily nonrenal.

### Comparative metabolism.

**FIG 2:**
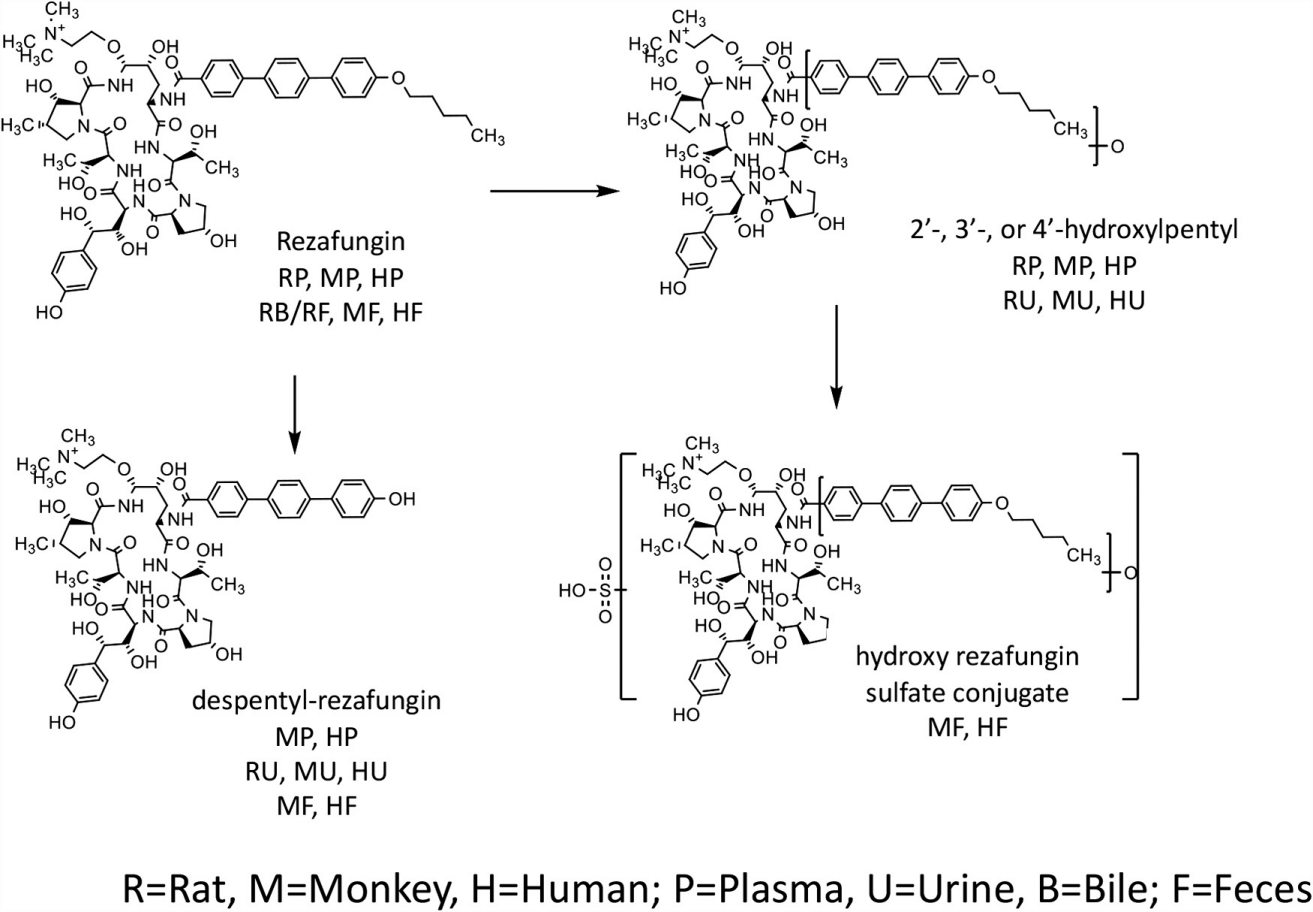
Scheme of rezafungin metabolism.

In general, rezafungin was metabolized by hydroxylation of the terphenyl, pentyl ether side chain in forming three hydroxylated metabolite isomers, namely, 2’-, 3′-, or 4’-hydroxylpentyl rezafungin. A second biotransformation observed involved the loss of the pentyl group via *O*-dealkylation to form metabolite despentyl-rezafungin (Fig. 2). Subsequent conjugation (sulfation) of the hydroxyl metabolites was observed to a minimal extent.

The metabolite profile of rat plasma samples showed primarily unchanged parent drug (56% of total plasma radioactivity exposure), although hydroxylated metabolites, 4’-hydroxylpentyl rezafungin and 2’-hydroxylpentyl rezafungin, were detected at low levels at later time points (≥ 4 h) and amounted to about 33% and 11% over the same time period, respectively. In rat feces and bile, unchanged parent drug was the predominant radioactive component. In contrast, rat urine comprised mainly hydroxylated metabolites, 4’-hydroxylpentyl rezafungin (62%), 3′-hydroxylpentyl rezafungin (1.9%) and 2’-hydroxylpentyl rezafungin (7.8%), as well as the *O*-dealkylated metabolite, despentyl-rezafungin (28% of total recovered radioactivity).

Similar to the rat, unchanged parent was the major circulating component (74% of total plasma radioactivity exposure) observed in monkey plasma with metabolites 4’-hydroxylpentyl rezafungin, 3′-hydroxypentyl rezafungin, and 2’-hydroxylpentyl rezafungin calculated to be 8.3%, 6.3%, and 3.0% over the same time period, respectively. In monkey feces, unchanged parent was the major radioactive component observed, representing 35% of the total recovered radioactivity with the observed highest metabolite, despentyl-rezafungin, representing 25% of total recovered radioactivity. Hydroxylated and subsequently sulfated hydroxyl metabolites accounted for 4% to 22% of total recovered radioactivity. Excretion in monkey urine comprised mainly hydroxylated metabolites, 4’-hydroxylpentyl rezafungin (42%), 3′-hydroxylpentyl rezafungin (12%), and 2’-hydroxylpentyl rezafungin (25%), as well as the *O*-dealkylated metabolite, despentyl-rezafungin (22% of total recovered radioactivity).

The primary metabolic pathways of rezafungin in humans reflected what was observed for the rat and monkey. Metabolism of rezafungin was localized predominantly to the pentoxy side chain and was mediated primarily by hydroxylation. *O*-dealkylation and sulfation were comparatively minor primary and secondary biotransformation pathways. In human plasma, rezafungin was the most abundant circulating component, calculated at 69% of the total plasma radioactivity exposure. Hydroxylated metabolites, 4’-hydroxylpentyl rezafungin, 3′-hydroxypentyl rezafungin, and 2’-hydroxylpentyl rezafungin were the most abundant circulating metabolites, comprising 9.8%, 5.5%, and 6.9% of total plasma radioactivity exposure, respectively. Unchanged rezafungin was the major radioactive component in human feces, accounting for 90% of total recovered radioactivity, while trace to minor amount of rezafungin metabolites cumulatively accounted for the rest. The hydroxy rezafungin sulfate conjugate was the most abundant, albeit minor, fecal metabolite and accounted for 4.3% of the total recovered radioactivity. Elimination of unchanged rezafungin in urine was negligible while metabolites, 4’-hydroxylpentyl rezafungin, 3′-hydroxypentyl rezafungin, 2’-hydroxylpentyl rezafungin, and despentyl rezafungin were the most abundant excreted in urine, comprising 49%, 6.2%, 28%, and 13% of the total recovered radioactivity in urine, respectively.

## DISCUSSION

The objective of these studies was to conduct definitive experiments to investigate the pharmacokinetics, metabolism, and mass balance of rezafungin in preclinical species used for safety testing in rats and monkeys, as well as in humans.

**FIG 3:**
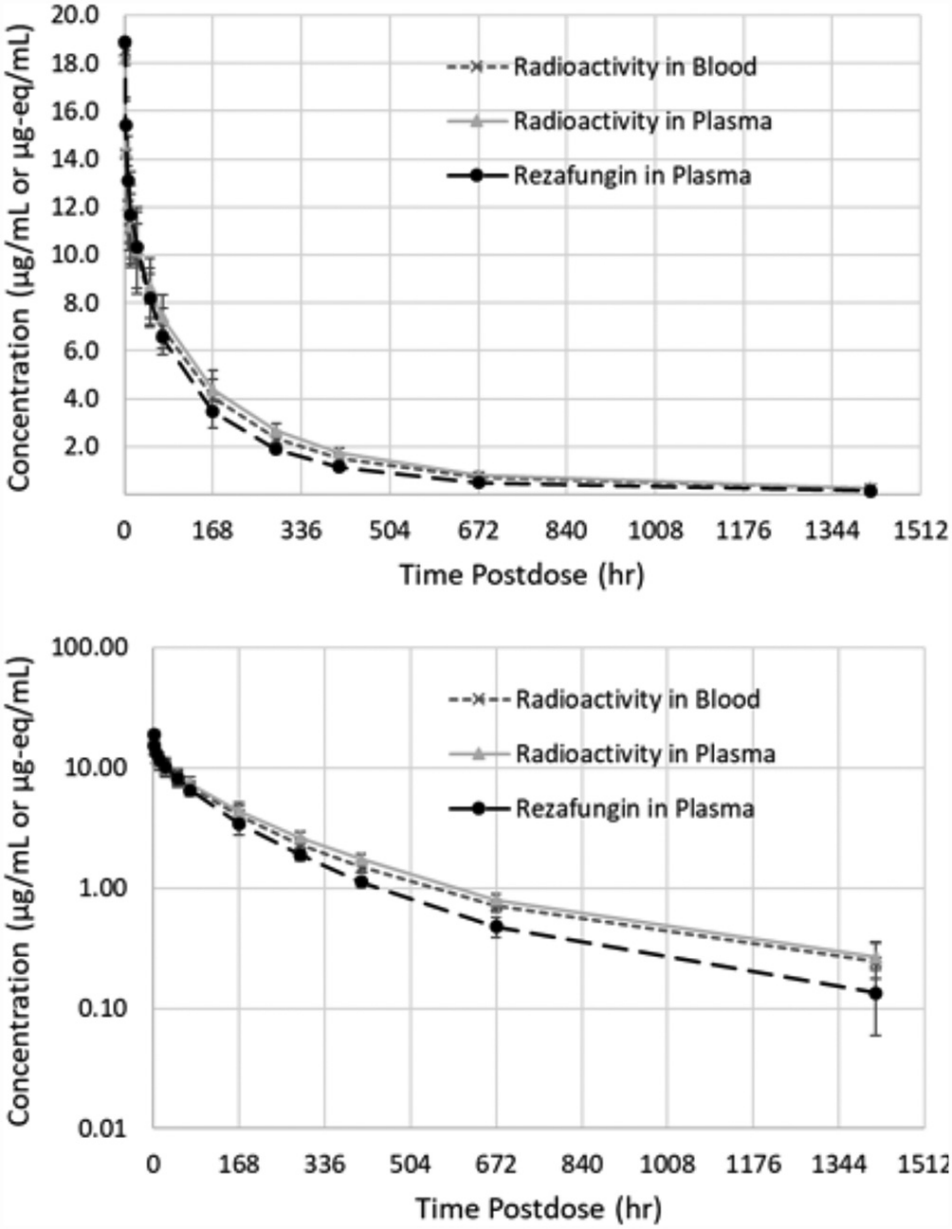
Mean concentration-time profiles (top: linear; bottom: semi-log) for whole blood/plasma radioactivity and plasma rezafungin for human subjects.

In animals and humans, plasma radioactivity profiles indicate very slow elimination following IV administration of [^14^C]-rezafungin. In the rat and monkey, mean plasma total radioactivity concentrations decreased slowly over time with t_1/2_ values of 54 h and 170 h, respectively. Calculated blood-to-plasma ratios were generally close to 1 and suggest approximately equal distribution of radioactivity in plasma and the cellular fraction of blood. An even slower elimination profile was observed in humans, as shown in Fig. 3 and Table 1, with plasma radioactivity t_1/2_ value of 387 h. Mean blood/plasma concentration ratios ranged from 0.86 to 1.0 through the last collection time point (day 60), confirming the observation in the rat and monkey of the low association of rezafungin with blood cells (Fig. 3).

**FIG 4:**
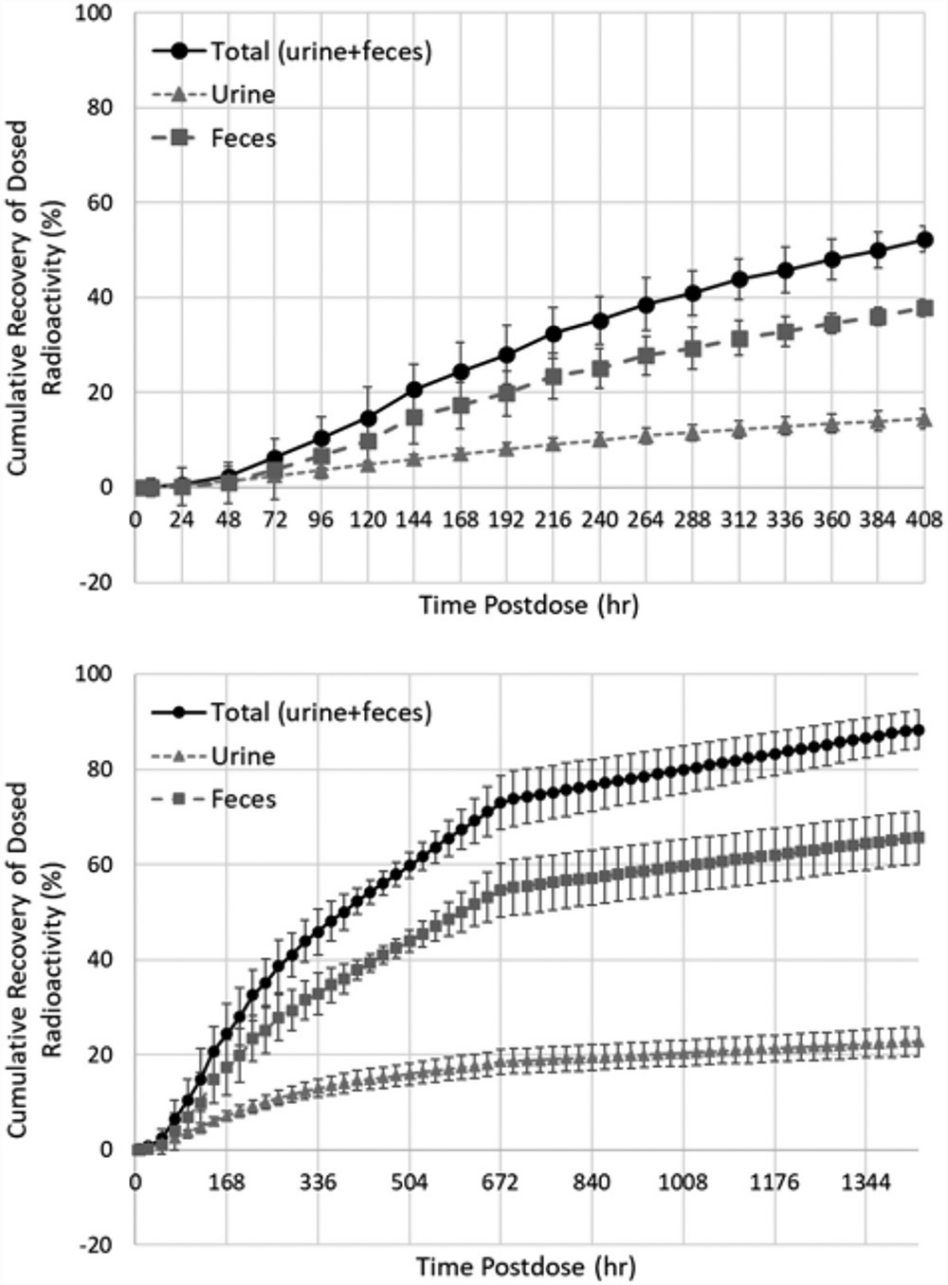
Cumulative recovery (mean ± standard deviation) of radioactivity from human excreta collected through the first 17 days (top) and by day 60 based on linear interpolation (bottom).

Preclinical ADME studies in rats and monkeys had shown that rezafungin is primarily excreted as unchanged drug in feces, with urinary excretion as a minor route (16). Across animals and humans, the fraction of the total recovered radioactive dose was generally 0.74 to 0.81 in the feces with sustained or prolonged elimination of radioactivity over time. For example, in humans, cumulative recovery of radioactivity from excreta collected through the first 17 days was 52% (38% in feces, 14% in urine), highlighting the slow overall elimination of rezafungin. Even by day 60 (based on linear interpolation), overall recovery of the administered dose was estimated to be less than complete at 88% (Fig. 4).

It was interesting to note that after IV dosing, [^14^C]-rezafungin was also observed in the feces, suggesting biliary excretion, intestinal secretion, passive diffusion, or some combination. Indeed, in bile duct cannulated rats, [^14^C]-rezafungin also predominated in the feces (35%) compared with bile (17%), suggesting a combination mechanism of slow elimination. Biliary excretion has been observed for caspofungin(17) and anidulafungin(18). In the case of anidulafungin, although uncommon, passive diffusion (up to 10% of dosed fraction) was reported for anidulafungin, with which rezafungin shares a remarkable structural similarity.

The majority of the total radioactivity in plasma/feces/bile was associated with parent drug, suggesting minimal biotransformation of rezafungin. In both preclinical and clinical urine samples, although unchanged drug was detected in the initial/early urine samples, hydroxylated metabolites were the predominant components in urine samples. These observations suggest that the rate of formation of metabolites is slow, and support the slower elimination of rezafungin overall.

All three of the previously approved echinocandins (caspofungin, micafungin, and anidulafungin) are believed to undergo spontaneous chemical (non-enzymatic) degradation to a ring-opened peptide as their primary clearance mechanism (18). In contrast, rezafungin with a quaternary ammonium side chain modification prevents the ring opening and enhances the stability of the parent molecule (12), allowing for an extensively prolonged plasma half-life compared with the three older echinocandins. While protein binding is generally considered a factor in evaluation of half-life, rezafungin and the three approved echinocandins all demonstrate comparable protein binding values (97.4% and 92.4%–99.9%, respectively)(12, 19).

**FIG 5:**
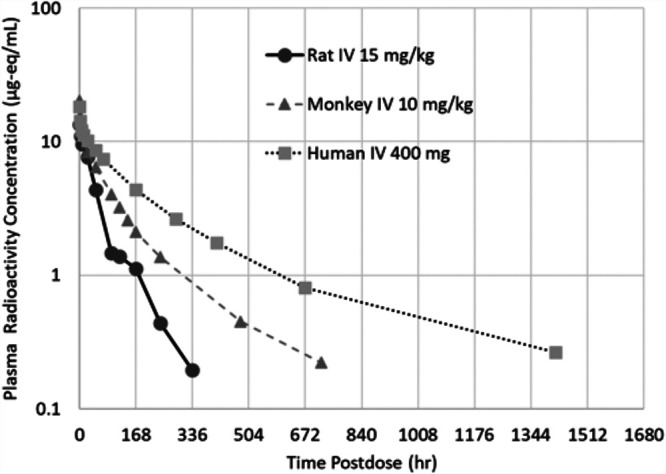
Plasma concentration-time profiles of plasma radioactivity for rat, monkey, and human.

The metabolism of rezafungin was qualitatively similar across both animal species examined (Fig. 5). In general, rezafungin was metabolized by hydroxylation of the terphenyl, pentyl ether side chain in forming three hydroxylated metabolite isomers, namely, 2’-, 3′-, or 4’-hydroxylpentyl rezafungin as well as loss of the pentyl group via *O*-dealkylation to form metabolite despentyl-rezafungin (scheme). The hydroxylated metabolites were more quantifiable in monkey plasma, with the largest at about 8% of the total radioactivity as measured by AUC. Subsequent conjugation of the oxidative metabolites was observed to a minimal extent. In plasma and feces (or rat bile), rezafungin was the predominant compound measured. Rezafungin accounts for ∼77% of total radiocarbon AUC and metabolites accounted for less than 10% of the total plasma radioactivity AUC exposure, as shown in the concentration-time profiles. In the urine, as observed in rat and monkey metabolite profiling studies, low level, inactive, oxidative metabolites were identified as 2’-, 3′-, 4’-hydroxylpentyl rezafungin, and despentyl-rezafungin.

A similar metabolism profile was observed in human samples, in which parent drug was predominant. Unsurprisingly, results from the human excretion/mass balance, metabolism, and PK study are consistent with nonclinical results, which showed fecal excretion as the major route of elimination of [^14^C]-rezafungin. The effect of the low level presence of rezafungin in intestinal microbiota is currently unknown and bears further study, although we do note that both caspofungin and anidulafungin undergo biliary excretion, implying that a small fraction of the dose is continually present in the intestinal tract and eliminated as unchanged drug in the feces(17, 18). Overall, rezafungin underwent minimal metabolism and slow elimination after an intravenous administration. In conclusion, rezafungin displays similar disposition and metabolism properties in preclinical species and in humans.

## MATERIALS AND METHODS

All studies that involved animals were approved by an Institutional Animal Care and Use Committee and were conducted in compliance with appropriate local health regulations and ethical approval.

### Chemicals.

For the rat and monkey mass balance study, [^14^C]-rezafungin (46.53 μCi/mg; radiochemical purity, 99.6%) was synthesized by Moravek (Brea, CA) as a bulk powder, which was compounded with unlabeled rezafungin manufactured by Bachem Americas, Inc. (Torrance, CA) as a solution in a vehicle of 2.5% Tween 80, 5% mannitol, 0.3% glacial acetic acid adjusted to pH 4.5.

For the human mass balance study, [^14^C]-rezafungin (5.7 μCi/mg; radiochemical purity, 94.3%) was synthesized by ViTrax (Placentia, CA) as a bulk powder, which was compounded with unlabeled rezafungin manufactured by Bachem Americas (Torrance, CA).

**FIG 6:**
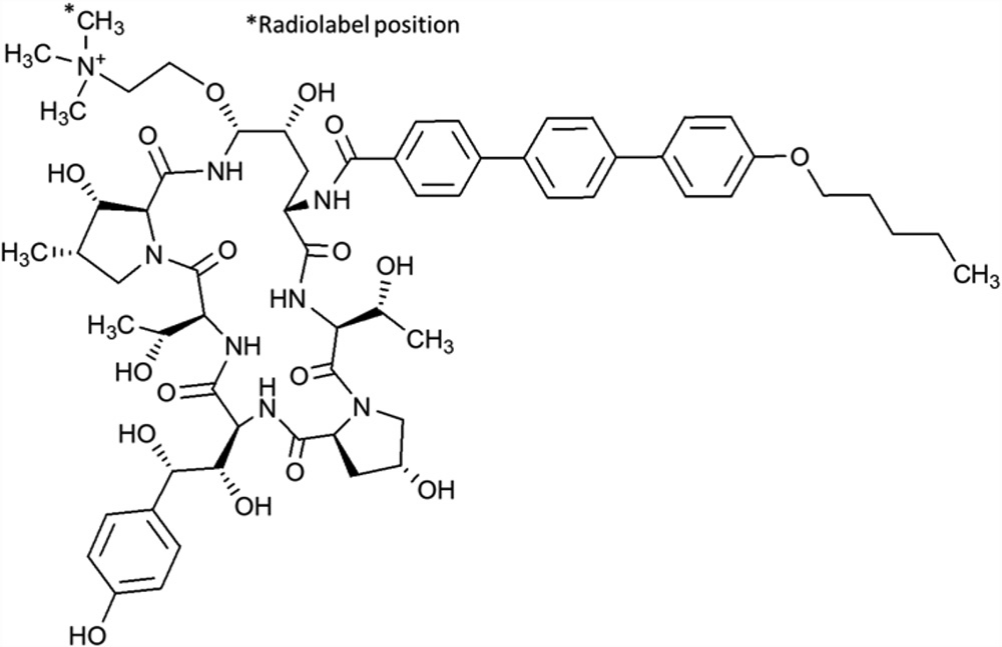
Position of radiolabel on [^14^C]-rezafungin.

The chemical structure of rezafungin with the radiocarbon position is depicted in Fig. 6. Chemical standards of the hydroxylated (2’-, 3′-, and 4’-hydroxy) and despentyl metabolites of rezafungin were synthesized and supplied by Hypha (Oxfordshire, UK). Chemicals, reagents, and HPLC grade solvents were obtained from commercial sources.

### Rat/monkey mass balance.

**(i) Rat study design.** The study used 12 male Sprague-Dawley rats, each administered a bolus IV injection into the tail vein over approximately 3–4 min of 15 mg/kg of rezafungin (with a target radioactivity of 200 μCi/kg of [^14^C]-rezafungin). Three of the rats were bile duct cannulated. Whole blood/plasma, urine, feces/bile, and cage washings were collected at periodic intervals from noncannulated rats until euthanization at 336 h postdose and from bile duct cannulated rats until euthanization at 120 h postdose as that was the duration that the patency of bile duct cannulae could be maintained. Carcasses were collected at the time of euthanization. All samples were analyzed for drug-derived radioactivity by liquid scintillation counting.

**(ii) Monkey study design.** The study used 3 male cynomolgus monkeys, each administered a single 10 mg/kg IV dose of [^14^C]-rezafungin (with a target radioactivity of 100 μCi/kg of [^14^C]-rezafungin) by infusion into a saphenous vein over 20 min. Whole blood and plasma samples were collected at each of the following time points: predose, 0.5, 2, 4, 8, 24, 48, 96 120, 144, 168, 240, 480, 720, and 1440 h postdose. Urine, feces, and cage washings were collected at predose (urine/feces only) and daily 24-h collections for 30 days.

**(iii) Sample analysis.** Whole blood and fecal homogenates were combusted prior to analysis by liquid scintillation counting (LSC). Blood, plasma, bile, urine, cage rinse/wash, and fecal homogenate samples were mixed with scintillation fluid and analyzed for radioactivity by LSC. Rat carcasses were dissolved in potassium hydroxide, and the resulting digests were mixed with scintillation fluid and analyzed for radioactivity by LSC.

**(iv) Metabolite profiling.** The metabolite identification and urine profiling were achieved using pooled samples by equal percent weight of samples (urine, bile, feces) or by equal volume (50 μl) aliquots of each plasma sample across all time points/intervals. The urine, plasma, bile, and fecal samples were analyzed using liquid chromatography tandem mass spectrometry (LC-MS) coupled with a radioactivity flow-through detector for metabolite profiling and identification. Tandem MS experiments were performed, and the structures of the metabolites were proposed based on their MS/MS spectra. The proposed structures were confirmed by comparison of the retention times and product ion spectra with the authentic reference standards when possible.

### Human mass balance.

**(i) Study design.** This was an open-label, phase I, single-dose mass balance study. The study was conducted at Labcorp (formerly, Covance, Inc.; Madison, WI) in accordance with good clinical practice, including ICH guidelines and applicable regulatory requirements, and in general conformity with the Declaration of Helsinki. No animal work was conducted by Labcorp. All pertinent study documents were reviewed by the Institutional Review Board (IRB) prior to study initiation. Following IRB approval and collection of written informed consent, all the subjects underwent an initial screening assessment within 28 days of the first dose. Safety was assessed by vital signs, physical examinations, AE assessments, laboratory tests (chemistry, hematology, and urinalysis), and a 12-lead electrocardiogram.

Subjects were initially confined in the clinical research unit (CRU) for 17 days postdose and returned for two follow-up visits (days 29 and 60). Recovery of radioactivity was estimated by linear interpolation during the period of time subjects were away from the CRU. Blood samples, urine, and feces were collected at specified times over 60 days.

**(ii) Sample analysis.** Whole blood, plasma, urine, and fecal homogenate samples were mixed with scintillation fluid and analyzed by liquid scintillation counting. For non-radiolabeled rezafungin concentration determination, a bioanalytical method was validated for measuring rezafungin in human plasma that is similar to one previously reported for animal plasma (16). Samples were analyzed using a 100 μl aliquot volume and a protein-precipitation extraction procedure followed by liquid chromatography/tandem mass spectrometry (LC-MS/MS) analysis. Chromatography was carried out on a Waters Atlantis HPLC column (dC18, 2.1 × 50 mm, 5 μm) operating under reverse-phase gradient conditions (0.1% formic acid in water/0.1% formic acid in acetonitrile/tetrahydrofuran ramped from 55%/45%/0% to 5%/0%/95%). Rezafungin calibration range was 10.0 ng/ml to 10,000 ng/ml using isotopically-labeled *d*9-rezafungin as an internal standard. An AB-Sciex API 4000 mass spectrometer was operated in the multiple reaction monitoring mode (parent-to-product ion transition) under optimized conditions for detection of rezafungin (*m/z* 604.5 to 343.3 Da) and *d*9-rezafungin (*m/z* 609.5 to 343.3 Da) positive ions formed by electrospray ionization. Excellent intra- and inter-assay accuracy and precision, as measured by quality control (QC) samples, was obtained during validation. Across the range of QC samples tested (lower limit of quantitation, LLOQ, through the High QC range), the intra-assay accuracy (% bias from nominal) ranged from −1.4% to 7.0% with the intra-assay precision (% coefficient of variation) ranging from 1.6% to 7.4%. Inter-assay accuracy ranged from 0.3% to 4.3% with the inter-assay precision ranging from 2.7% to 5%.

**(iv) Metabolite profiling.** Plasma samples were pooled across subjects through 672 h postdose to produce inter-subject time point pools. Urine and fecal homogenate samples were pooled by subject across various time points up through 408 and 672 h postdose, respectively. Selected samples of plasma, urine, and feces were profiled for rezafungin and metabolites by HPLC with radiochemical and high-resolution mass spectrometry detection; metabolites were identified by HPLC with known standards and/or by HPLC-MS/MS structure elucidation. Pharmacokinetics were calculated by non-compartmental analysis using Phoenix WinNonlin software (version 6.3/8.1; Pharsight, Mountain View, CA).
